# Controlled Photon Switch Assisted by Coupled Quantum Dots

**DOI:** 10.1038/srep11169

**Published:** 2015-06-22

**Authors:** Ming-Xing Luo, Song-Ya Ma, Xiu-Bo Chen, Xiaojun Wang

**Affiliations:** 1 Information Security and National Computing Grid Laboratory, Southwest Jiaotong University, Chengdu 610031, China; 2 School of Mathematics and Statistics, Henan University, Kaifeng 475004, China; 3 State Key Laboratory of Networking and Switching Technology, Beijing University of Posts and Telecommunications, Beijing 100876, China; 4 School of Electronic Engineering, Dublin City University, Dublin 9, Ireland

## Abstract

Quantum switch is a primitive element in quantum network communication. In contrast to previous switch schemes on one degree of freedom (DOF) of quantum systems, we consider controlled switches of photon system with two DOFs. These controlled photon switches are constructed by exploring the optical selection rules derived from the quantum-dot spins in one-sided optical microcavities. Several double controlled-NOT gate on different joint systems are greatly simplified with an auxiliary DOF of the controlling photon. The photon switches show that two DOFs of photons can be independently transmitted in quantum networks. This result reduces the quantum resources for quantum network communication.

Photonic schemes are very important in quantum information processing because of their superiority on the speed^1^. However, it is not easily to realize deterministic all-optical quantum gates based on single photons. The difficulty to achieve photon-photon interactions as a major challenge also exists in experimental quantum networks, which are connected by material quantum nodes interconnected by photonic channels^2^,^3^,^4^. One primitive element in these architectures is the efficient switching and routing of photons^5^,^6^. The photonic switching may be actuated by optically induced refractive index changes, and the switching speed is limited by the free carrier generation^7^,^8^. Other approaches employ silicon-organic hybrid waveguides for very fast signal processing^9^ or slow light in coupled photonic crystal waveguides for all-optical switching^10^.

Recently strong quantum light-matter couplings in photonic nanostructures can produce effective interactions between photons, which have leaded some remarkable phenomena such as the photon blockade^11^,^12^, optical transistors^13^,^14^, and photonic quantum gates^15^. By carefully tailoring the local optical mode density, coherent and incoherent non-classical light can be distributed on a chip into a quantum photonic circuit^16^. Accordingly, considerable efforts have been made in recent decades towards photon-photon interactions using the mediation of material systems. The pioneered efforts may be the strong coupling between single atoms and optical microresonators by the cavity quantum electrodynamics (cQED)^17^,^18^,^19^. Based on the scheme^20^, a series of works have been made to achieve the nondestructive measurement of an optical photon^21^,^22^,^23^, single photon phase switching^24^, and the realizations of a quantum gate between flying photons and a single atom^25^, all of which may be applied to photon switching^26^,^27^,^28^,^29^,^30^,^31^,^32^,^33^.

In comparison to these results using one degree of freedom (DOF)^11^,^12^,^13^,^14^,^15^,^16^,^17^,^18^,^19^,^20^,^21^,^22^,^23^,^24^,^25^,^26^,^27^,^28^,^29^,^30^,^31^,^32^,^33^, an extensive amount of researches have focused on generating entanglement in one degree of freedom (DOF), such as the quadrature^34^, polarization^35^,^36^ or spatial field variables^37^,^38^. With these states even the generations of multimode entangled beams are possible, which may potentially simplify quantum communication systems, especially if multiple modes are contained within a single beam^39^. Manipulating the quantum mechanical properties of more than one DOF has already been demonstrated as hybrid- and hyper-entanglement^40^,^41^,^42^,^43^,^44^,^45^ have been thoroughly investigated. In order to take the next step towards scalable quantum networks, there is a need for phonon switching schemes because multiple degrees of freedoms are compatible with photonic circuits simultaneously^46^,^47^,^48^.

In this paper, we consider phononic switching schemes of two DOFs of photon states using the optical circular birefringence of a one-sided QD-cavity system. Most previous results^26^,^27^,^28^,^29^,^30^,^31^,^32^,^33^ are related to the switching on one DOF of quantum systems, such as the polarization DOF of photon systems. Generally, one DOF (spatial-mode DOF) may assist quantum logic gates performed on the other DOF (polarization DOF)^49^,^50^,^51^,^52^,^53^. We investigate the possibility of parallel quantum transmissions of two DOFs of photon systems. All switching schemes may be controlled by photon or stationary electron spins in quantum dots^54^,^55^,^56^,^57^,^58^,^59^,^60^. For simplification of the implementations, the deterministic hyper controlled-NOT gates and auxiliary DOF of the controlling photon are used to realize deterministic switches of the spatial-mode and the polarization DOFs of a two-photon system. These results are beyond the switching gates on the same DOF of two-photon state^26^,^27^,^28^,^29^,^30^,^31^,^32^,^33^ and realization of the Toffoli gate^53^. The primitive schemes are also adapted to multiport switching with an improved quantum routing. Our theoretical results show that two DOFs of photon systems can be used as independent qubits in quantum network communication.

## Results

### Controlled quantum switch

**Figure 1:**
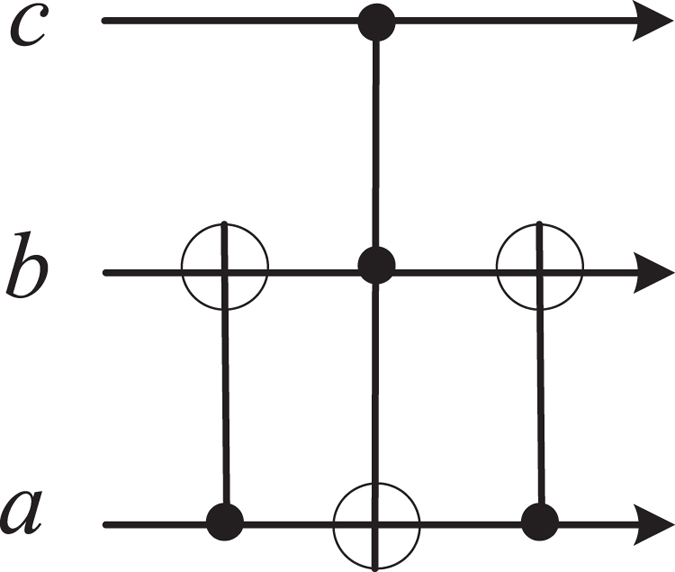
Schematic controlled 2 × 2 quantum switch. Two CNOT gates and one Toffoli gate are used. *a* and *b* are input qubits of one switch while *c* is the controlling qubit. The switching operation is implemented if *c* is in the state 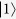.

The primitive block of the proposed reconfigurable quantum switch is the controlled 2 × 2 quantum swapping gate^6^,^26^,^27^,^28^,^29^,^30^,^31^,^32^,^33^ for three qubit states *a*, *b* and *c*, shown in Fig. 1(b). The input qubits *a* and *b* may be swapped if the qubit *c* is 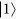. Otherwise, *a* and *b* are unchanged. The hyper-photons *a* and *b* with the polarization and spatial-mode DOFs are considered in this paper. Our motivation is to manipulate them simultaneously. Thus these DOFs may be applied as independent qubits in quantum information processing. Different from the detailed decomposition of the Toffoli gate with six CNOT gates^53^, it may be greatly simplified with one auxiliary DOF of the controlling photon and auxiliary spins. Since each DOF of the photon may play different roles in a quantum switching, four different quantum switchings are considered, i.e., two circuits for switching the same DOF of two photons while two circuits for switching different DOF of two photons^54^,^55^,^56^,^57^,^58^,^59^,^60^. None of them requires changing photon DOFs during transmissions. From these primitive quantum switchings, general multiport quantum switchings may be easily constructed for photon systems. It means that each DOF of photon systems can be viewed as an independent qubit in quantum network communications.

### Quantum dot system

**Figure 2:**
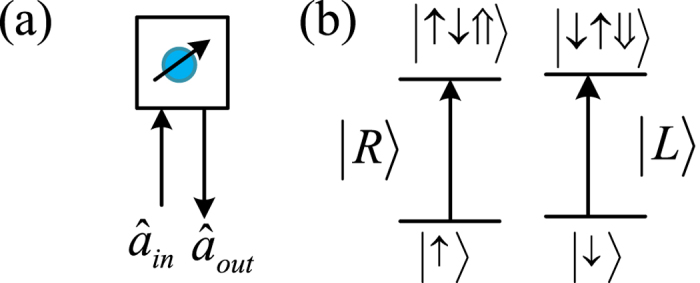
Schematic dipole spin-dependent transitions with circularly polarized photons. (**a**) A charged QD inside a one-side micropillar microcavity interacting with circularly polarized photons. *â*_*in*_ and *â*_*out*_ are the input and output field operators of the waveguide, respectively. (**b**) dipole spin-dependent optical transition rules due to the Pauli exclusion principle. 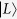 and 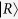 represent the left and right circularly polarized photon respectively. 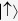 and 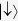 represent the spins of the excess electron. 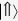 and 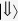 describe the heavy-hole spin states 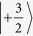 and 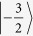 respectively.

To complete controlled quantum switches of hyper photons, the following optical property and quantum dot system (QD) are used for our schemes^48^,^49^,^54^,^55^,^56^,^57^,^58^,^59^,^60^. The QD-cavity system is constructed by a singely charged QD [a self-assembled In(Ga)As QD or a GaAs interface QD] located in the center of a one-sided optical resonant cavity, as shown in Fig. 2. For the excess electron-spin state 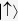 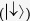, a negatively charged exciton 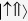 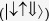 with two antiparallel electron spins^60^ is generated by resonantly absorbing 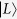 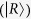. From the Heisenberg equations^60^ of the cavity field operator and dipole operator, the QD likes a beam splitter with the reflection coefficient


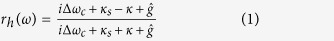


if the dipole stays in the ground state at most of the time^49^,^54^,^55^,^56^,^57^,^58^,^59^,^60^ [the signs of *κ*_*s*_ and *κ* should be changed^49^, i.e., 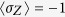. Here, 
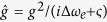
, Δ*ω*_*c*_ = *ω*_*c*_ − *ω* and Δ*ω*_*e*_ = *ω*_*e*_ − *ω*. *ω*_*c*_, *ω*_*e*_ and *ω* are the frequencies of the cavity mode, the input probe light, and the dipole transition, respectively. *g* is the coupling strength between the cavity and dipole. *ς*, *κ*, and *κ*_*s*_ are the decay rates of the dipole, the cavity field, and the cavity side leakage mode, respectively. The reflection coefficient in equation (1) becomes


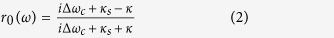


if the QD is uncoupled from the cavity (*g* = 0)^49^,^61^. Thus by adjusting *ω* and *ω*_*c*_, the reflection coefficients can satisfy 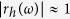 and 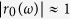 when the cavity side leakage is negligible. If one photon in the state 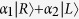 enters into a one-sided QD system with the spin state 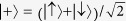, the joint system of the photon and spin after reflection is


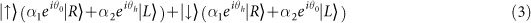


where 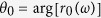 and 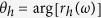. By adjusting *ω* and *ω*_*c*_, one can get *θ*_0_ = *π* and *θ*_*h*_ = 0. It follows an optical selection rule^49^,^54^


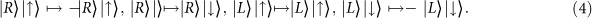


Based on these optical selection rules, the following CNOT gates^49^ may be implemented on two photons *x* and *y* with two DOFs


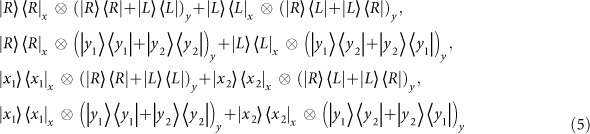


where 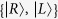 is the basis of the polarization DOF while 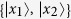 and 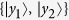 are the bases of the spatial mode DOF of the photons *x* and *y* respectively.

### Controlled photon switch

**Figure 3:**
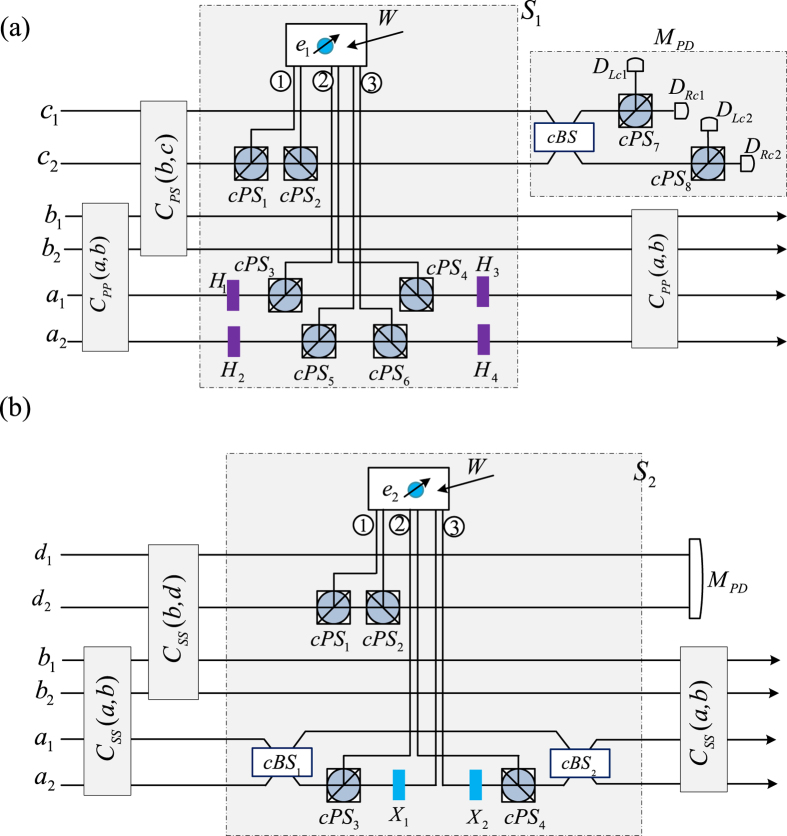
Controlled photon switch. (**a**) Controlled switch of two polarization states. *H*_*j*_ represents a half-wave plate to perform the Hadamard operation on the polarization DOF of a photon. *cBS*_*j*_ represent 50%50 beam splitters to perform the Hadamard operation on the spatial DOF of a photon. *cPS*_*j*_ represent circulated polarization beam splitters to transmit 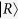 and reflect 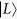. The CNOT gates *C*_*PP*_(*a*, *b*) and *C*_*PS*_(*b*, *c*) are defined in the equation (5). This circuit implements the controlled swapping the polarization DOFs of the photons *a* and *b*. The spatial mode of the photon *c* is an auxiliary qubit. (**b**) Controlled switch of two spatial mode DOFs. The CNOT gate *C*_*SS*_(*x*, *y*) is defined in the equation (5). *X*_*i*_ represent wave plates to perform Pauli flip *X* on the polarization DOF of a photon. *W* represents the Hadamard gate on the spin. This circuit implements the controlled swapping the spatial modes of the photons *a* and *b*. The bold line denotes the controlling photon. *e*_*i*_ are auxiliary spins in the state 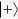.

Using the CNOT gates in the equation (5) [on the two DOFs of two photons^49^] and the switch circuit in the Fig. 1, two photons’ switch may be controlled, as shown in Fig. 3. Assume that the photons *a* and *b* in the states


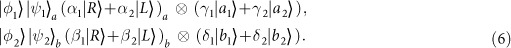


The input states in Fig. 3(a) are the polarization qubits of two photons *a* and *b* while the spatial mode DOFs of two photons *a* and *b* are presented in Fig. 3(b). In order to simplify the implementation of the double controlled NOT gate, the controlling photons *c* and *d* are also photons in the state 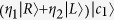 and 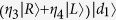 for generality. Here, 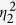 and 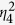 denote the switching probabilities.

From the Fig. 3(a), after the CNOT gate *C*_*PP*_(*a*, *b*) on the polarization DOF of two photons *a* and *b*, the joint system of the photons *a* and *b* becomes


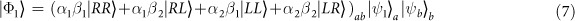


From Fig. 1, if the polarization DOF of the photon *c* is in the state 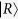, the controlled-CNOT gate (Toffoli gate) does not fire. If the polarization DOF of the photon *c* is in the state 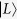, the Toffoli gate fires. Generally, in order to simplify the Toffoli gate on the polarization DOF of three photons, the auxiliary spatial mode DOF of the photon *c* and an auxiliary spin *e*_1_ in the state 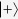 are used. After the CNOT gate *C*_*PS*_(*b*, *c*) in equation (5) [on the polarization DOF of the photon *b* and the spatial mode DOF of the photon *c*], the joint system of three photons *a*, *b*, and *c* is changed into


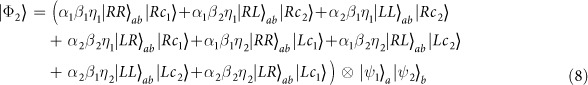


And then, let the photon *c* from the spatial mode *c*_2_ pass through the *cPS*_1_, the cavity Cy_1_ [with *e*_1_], and *cPS*_2_ from the path ①. The state 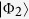 and the spin *e*_1_ are changed into


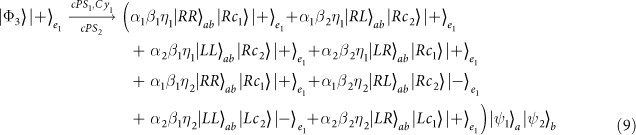


which may be transformed into


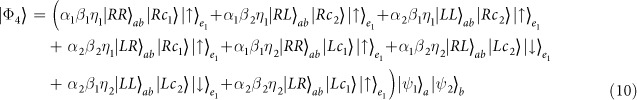


by performing a Hadamard operation *W* on the spin *e*_1_. Now, the photon *a* from the spatial mode *a*_1_ passes through the *H*_1_, *cPS*_3_, the cavity *Cy*_1_, *cPS*_4_, and *H*_3_ from the path ②, while the photon *a* from the spatial *a*_2_ passes through the *H*_3_, *cPS*_5_, the cavity *Cy*_1_, *cPS*_6_, and *H*_4_ from the path ③. The the joint system 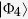 is transformed into


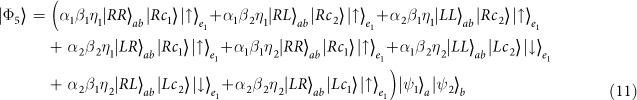


Moreover, measures the spin *e*_1_ under the basis 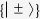 and the Pauli phase flip *Z* is performed on the photon *c* from the spatial mode *c*_2_ for the measurement outcome 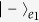. Thus 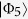 collapses into


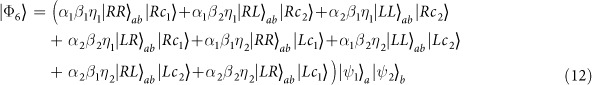


Furthermore, after performing the CNOT gate *C*_*PP*_(*a*, *b*) on the polarization DOF of the photons *a* and *b*, 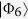 changes into


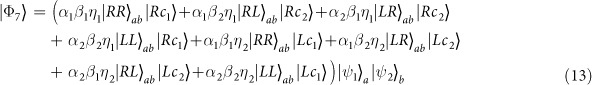


Finally, measures the photon *c* under the basis 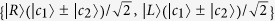 realized with the *cBS*_2_, *cPS*_7_, *cPS*_8_ and four single photon detectors. If the photon *c* is detected at 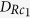 or 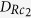 with the total probability 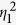, then 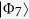 collapses into


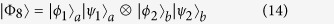


where one phase flip *Z* is performed on the polarization DOF of the photon *a* and *b* for 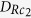. It means that the photons *a* and *b* have not been switched, i.e., the Toffoli gate is not fired. Otherwise, the photon *c* is detected at 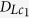 or 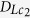 with the total probability 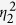, and 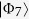 collapses into


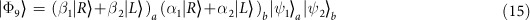


where one phase flip *Z* is performed on the polarization DOF of the photon *a* and photon *b* for 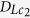. The polarization DOF of the photons *a* and *b* have been switched, i.e., the Toffoli gate is fired. the controlled quantum switch on the polarization DOF of two photons has been realized up to the specific assumption of the controlling photon *c*.

Similarly, the Fig. 3(b) presents the controlled quantum switch of spatial mode DOF of two photons *a* and *b*. The CNOT gate *C*_*SS*_(*a*, *b*) in the equation (5) [on the spatial mode DOF of two photons *a* and *b*^49^] is used to change the photons *a* and *b* into


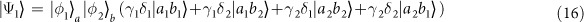


If the polarization DOF of the controlling photon *d* is 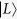, the Toffoli gate fires. In order to simplify the Toffoli gate on the polarization DOF of one photon and the spatial DOF of two photons, the auxiliary spatial mode DOF of the photon *d* and an auxiliary spin *e*_2_ in the state 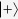 are used. In detail, the CNOT gate *C*_*SS*_(*b*, *d*) is performed on the photons *b* and *d* to get


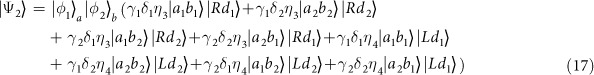


Let the photon *d* from the spatial mode *d*_2_ pass through the *cPS*_1_, the cavity Cy_2_ [with *e*_2_], and *cPS*_2_ from the path ①. 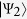 and the spin *e*_2_ are changed into


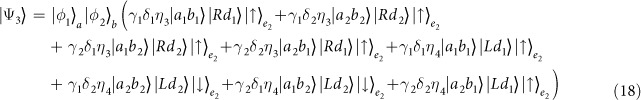


after a Hadamard operation *W* performed on the spin *e*_2_. Now, the *cBS*_1_ is used to realize a Hadamard operation on the spatial mode DOF of the photon *a*. And then, the photon *a* from the spatial mode *a*_2_ passes through the *cPS*_3_, where the reflected part passes through the cavity *Cy*_2_ from the path ② while the transmitted part passes through the *X*_1_, the cavity *Cy*_2_, and *X*_2_ from the path ③, all of them combined into one photon from the *cPS*_4_. Thus 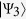 becomes


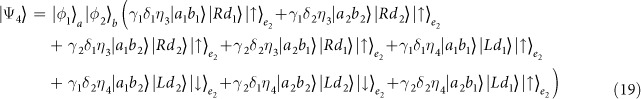


after the output photon *a* passing through the *cBS*_2_. The spin *e*_2_ is disentangled by measuring it under the basis 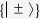 . 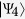 collapses into


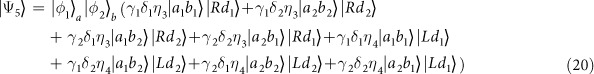


where the Pauli phase flip *Z* is performed on the photon *d* from the spatial mode *d*_2_ for the measurement outcome 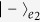. Moreover, the CNOT gate *C*_*SS*_(*a*, *b*) [on the spatial mode DOF of the photons *a* and *b*] may change 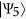 into


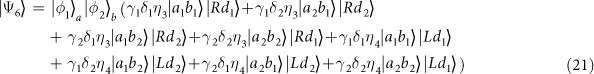


Finally, measure the controlling photon *d* under the basis 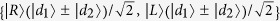 realized as *M*_*PD*_ in the Fig. 3(a). If the photon *d* is detected at 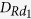 or 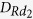 with the total probability 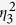, 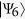 collapses into


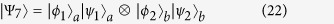


where the phase operation −*I* is performed on the polarization DOF of the photon *a* from the spatial mode *a*_2_ and −*I* is performed on the polarization DOF of the photon *b* from the spatial mode *b*_2_ for 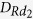. It shows that the photons *a* and *b* have not been switched, i.e., the Toffoli gate is not fired. If the photon *d* is detected at 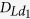 or 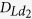 with the total probability 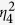, 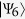 may collapse into


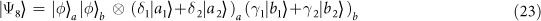


with the similar recovery operations for 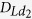. Thus, the spatial mode DOF of the photons *a* and *b* have been switched, i.e., the Toffoli gate is fired. Therefore, the controlled quantum switch of the spatial mode DOF of two photons has been realized up to the general assumption of the controlling photon *d*.

### Controlled cross switch of photons

**Figure 4:**
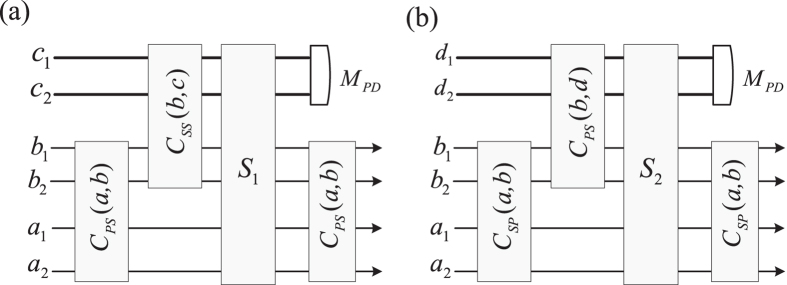
Controlled cross switch of photons. (**a**) Controlled polarization-spatial DOF switch. This circuit implements the controlled swapping of the polarization DOF of the hyper photon *a* and the spatial mode DOF of the hyper photon *b*. The CNOT gate *C*_*SP*_(*x*, *y*) is defined in the equation (5) [on the spatial mode DOF of the photon *x* and the polarization DOF of the photon *y*]. The subcircuit *S*_1_ is defined in the Fig. 3(a). *M*_*PD*_ denotes the measurement of the photon *c* defined in the Fig. 3. (**b**) Controlled spatial-polarization DOF switch. This circuit implements the controlled swapping of the spatial DOF of the photon *a* and the polarization DOF of the photon *b*. The subcircuit *S*_2_ is defined in the Fig. 3(b).

Derived from the circuit in the Fig. 3, different DOFs of photons may be switched under the controlling of one photon, shown in Fig. 4. All the input states in Fig. 4 are different DOFs of two photons *a* and *b*. The initial states of four photons *a*, *b*, *c* and *d* are same to these defined in the Fig. 3.

From the Fig. 4(a), after the CNOT gate *C*_*PS*_(*a*, *b*) on the polarization DOF of the photon *a* and the spatial mode DOF of *b* [shown in the equation (5)], the photons *a* and *b* are changed into


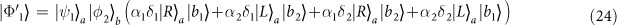


Now, if the polarization DOF of the photon *c* is 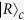 , the Toffoli gate does not fire while the Toffoli gate fires for 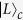. Similar to the quantum switch in the Fig. 3(a), the auxiliary spatial mode DOF of the controlling photon *c* and an auxiliary spin *e*_1_ in the state 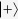 are used to simplify the hybrid Toffoli gate on two polarization qubits and one spatial qubit. In detail, the CNOT gate *C*_*SS*_(*b*, *c*) on the spatial mode DOF of two photons *b* and *c* [shown in the equation (5)] is used to change three photons *a*, *b*, and *c* into


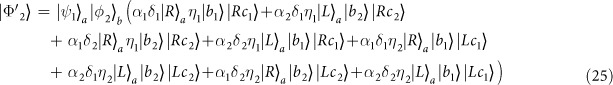


Similar to the Fig. 3(a), by using the auxiliary spin *e*_1_ in the state 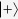, from the equations (8, 9, 10, 11, 12) the subcircuit *S*_1_ has realized the controlled gate


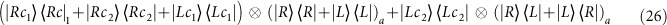


on the photon *c* and the polarization DOF of the photon *a*. Thus after this subcircuit, 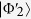 is transformed into


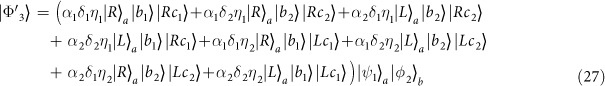


Moreover, using the CNOT gate *C*_*PS*_(*a*, *b*) on the polarization DOF of the photon *a* and the spatial mode DOF of the photon *b*, 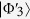 changes into


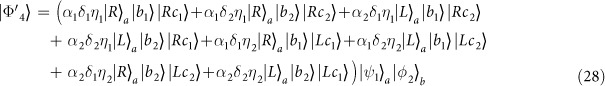


Finally, measure the controlling photon *c* using *M*_*PD*_ defined in Fig. 3(a). If the photon *c* is detected at 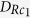 or 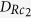 with the total probability 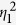, 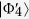 collapses into


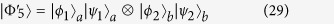


where the phase flip *Z* is performed on the polarization DOF of the photons *a* and *b* for 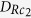. Two photons *a* and *b* have not been switched. Otherwise, the photon *c* is detected at 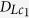 or 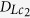 with the total probability 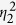, and 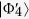 collapses into


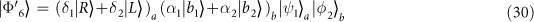


with the same recovery operation for 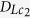. Thus, the polarization DOF of the photon *a* and spatial mode DOF of the photon *b* have been switched.

From the Fig. 4(b), the CNOT gate *C*_*SP*_(*a*, *b*) on the spatial mode DOF of the photon *a* and the polarization DOF of the photon *b* is used to change two photons *a* and *b* into


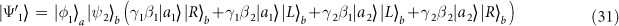


The followed Toffoli gate is controlled by the photon *d*. Similar to the quantum switch in the Fig. 3(b), the auxiliary spatial mode DOF of the controlling photon *d* and an auxiliary spin *e*_2_ in the state 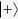 are used to simplify the hybrid Toffoli gate on two polarization qubits and one spatial qubit. In detail, the CNOT gate *C*_*PS*_(*b*, *d*) on the polarization DOF of the photon *b* and the spatial DOF of the photon *d* may change the three photons *a*, *b*, and *d* into


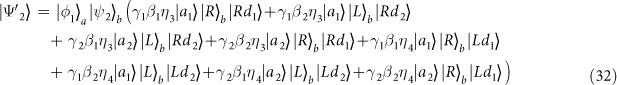


Similar to the Fig. 3(b), by using the auxiliary spin *e*_2_ in the state 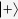, from the equations (17, 18, 19, 20) the subcircuit *S*_2_ has realized the controlled gate


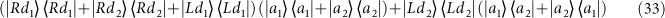


on the photon *d* and the spatial mode DOF of the photon *a*. After this subcircuit, 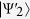 is changed into


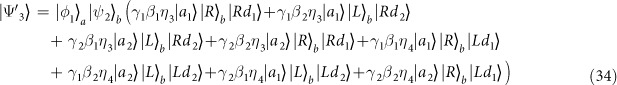


The followed CNOT gate *C*_*SP*_(*a*, *b*) [on the spatial mode DOF of the photon *a* and the polarization DOF of the photon *b* shown in the equation (5)] may change 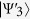 into


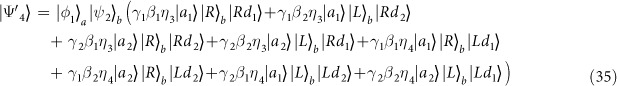


Finally, measures the photon *d* using *M*_*PD*_ defined in Fig. 3(a). If the photon *d* is detected at 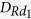 or 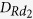 with the total probability 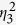, then 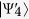 collapses into


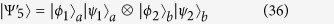


where one phase operation −*I* is performed on the photon *a* from the spatial mode *a*_2_ and the phase flip *Z* is performed on the polarization DOF of the photon *b* for 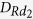. Otherwise, the photon *d* is detected at 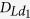 or 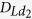 with the total probability 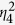, and 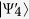 collapses into


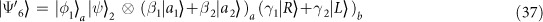


with the same recovery operations for 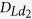. Thus, the spatial mode DOF of the photon *a* and polarization DOF of the photon *b* has been switched. Therefore, the different DOFs of two photons may be switched under the quantum control.

## Discussion

With ideal conditions, the cavity side leakage may be neglected, and the reflection coefficients are 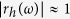 and 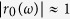. The corresponding fidelities of our switch circuit close to 100%. Unfortunately, the experimental fidelities may decrease because of the ruined transition rules in the equation (4) from the quantum decoherence and quantum dephasing. The imperfect spin-dependent transition rule decreases the fidelities by a few percent if the heavy-light hole mixing is considered. Fortunately, the hole mixing can be reduced by improving the shape, size, and type of QDs^22^. The neglect side leakage from the cavity should be considered in the experiment^21^,^22^,^58^,^59^,^60^,^61^. The electron spin decoherence may be also reduced by extending the electron coherence time to *μ*s using spin echo techniques^22^. The spin states 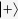 and 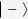 are generated using nanosecond electron spin resonance microwave pulses or picosecond optical pulses^60^, of which the preparation time (ps) is significantly shorter than the spin coherence time.

In the resonant condition *ω*_*c*_ = *ω*_*e*_ = *ω*, if the cavity side leakage is considered, the optical selection rules in the equation (4) become


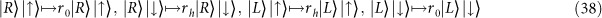


**Figure 5:**
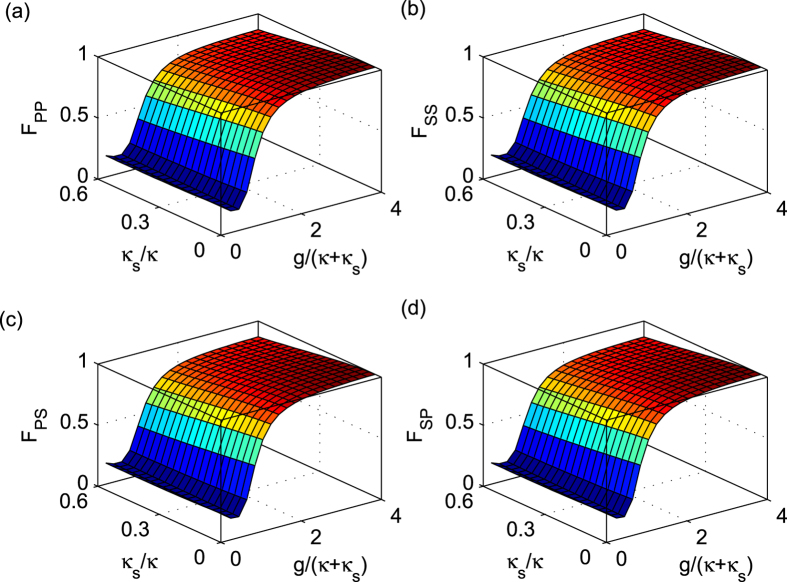
Average fidelities of the present photon switches. (**a**) The average fidelity *F*_*PP*_ of the polarization DOF switch on a two-photon system. (**b**) The average fidelity *F*_*SS*_ of the spatial DOF switch on a two-photon system. (**c**) The average fidelity *F*_*SP*_ of the photon cross switch on a two-photon system. (**d**) The average fidelity *F*_*PS*_ of the photon cross switch on a two-photon system. The coupling strength is defined by *ς* = 0.1*κ*_*s*_. The average fidelity is computed as the expectation of input photons. Here, 
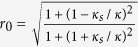
 from the equation (2) and *r*_*h*_ = 1 − [(1 + 3.64*g*^2^/(*κ* + *κ*_*s*_)^2^)^2^ + (1 + *κ*_*s*_/*κ* + 0.364*g*^2^/(*κ* + *κ*_*s*_)^2^)^2^]^−1/2^ from the equation (1).

The general fidelity is defined by 
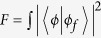
, where 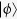 and 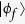 are final states under the ideal condition and experimental situation with side leakage respectively. Based on the optical selection rules in the equation (38), the fidelities of these four controlled quantum switches are evaluated in Fig. 5. Since these fidelities depend on the coefficients of the initial photons, they are presented as expectations of the initial states. From the Fig. 5, these average fidelities are very similar. There are several reasons. The first one is that all the CNOT gates [*C*_*PP*_(*x*, *y*), *C*_*PS*_(*x*, *y*), *C*_*SP*_(*x*, *y*), and *C*_*SS*_(*x*, *y*)] are only performed on the two-qubit states with different bases^49^ while other two qubits are unchanged. The second is that from the same optical rules in equation (38), all the CNOT gates [*C*_*PP*_(*x*, *y*),*C*_*PS*_(*x*, *y*),*C*_*SP*_(*x*, *y*), and *C*_*SS*_(*x*, *y*)] on different DOFs of the photons *x* and *y* lead to the same transformation on a four-dimensional space defined by the matrix


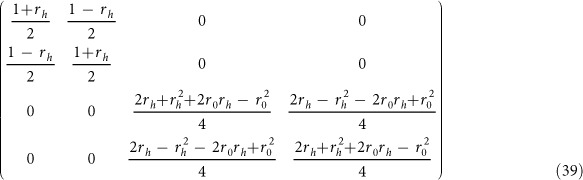


The differences are the spaces defined by the input qubits. The third is that the average fidelities are evaluated from the expectations of the initial states. Thus, the input qubit of the spatial DOF has no difference with the qubit of polarization DOF of each photon. The last is that the subcircuits *S*_1_ and *S*_2_ have realized the same double-controlled NOT gate shown in equation (26) and equation (33) respectively if the third reason is considered, i.e., the differences of two input qubits of the photon *a* are omitted. Generally, the high fidelity may be achieved from the strong coupling strength, the low side leakage and cavity loss rate *κ*_*s*_/*κ*. The strong coupling strength *g*/(*κ + κ*_*s*_) has been raised to 2.4 by improving the sample designs, growth, and fabrication^58^,^62^. When the coupling strength *g*/(*κ + κ*_*s*_) ≈ 2.4 with *κ*_*s*_/*κ *≈ 0, the fidelities of hyper photon switches are greater than 97.75%. In experiment, the side leakage and cavity loss rate have been reduced to *κ*_*s*_/*κ *≈ 0.7 with *g*/(*κ + κ*_*s*_) ≈ 1^57^,^58^. Recently, a quantum gate between the spin state of a single trapped atom and the polarization state of an optical photon contained in a faint laser pulse has been experimentally achieved^25^. We believe that their hybrid gate may be extended to our general hyper photon switches.

In conclusion, we have investigated the possibility of photon switches based on two DOFs of photon systems. By using several deterministic CNOT gates on the polarization and spatial mode DOF of a two-photon system and the simplified Toffoli gates on several three-photon systems, we design several controlled photon switches. Compared with the same DOF of photon switches^26^,^27^,^28^,^29^,^30^,^31^,^32^,^33^, our schemes have realized all possible switches of two DOFs of photon systems. Moreover, the controlling qubit may be chosen as a photon with two DOFs based on the hyper CNOT gates based on single-sided QD on a two-photon system^49^. Thus, our schemes are very convenience for quantum network communication based on photons with two DOFs because each DOF of photon may be applied without changing the DOF during transmissions. Compared with the photon switches^26^,^27^,^28^,^29^,^30^,^31^,^32^,^33^ with eight photonic CNOT gates [six for the Toffoli gate^53^], our circuits have only cost four CNOT gates on the two-photon system with the help of the auxiliary DOF of the controlling photon. Of course, the photon switches may be affected by the cavity leakage, and spin coherence in quantum dots or the exciton coherence in experiment. From experimental QD systems^57^ and hybrid controlled phase flip^25^, our switches are expected to be realizable for quantum network communication.

## Methods

### Parallel route-finding

**Figure 6:**
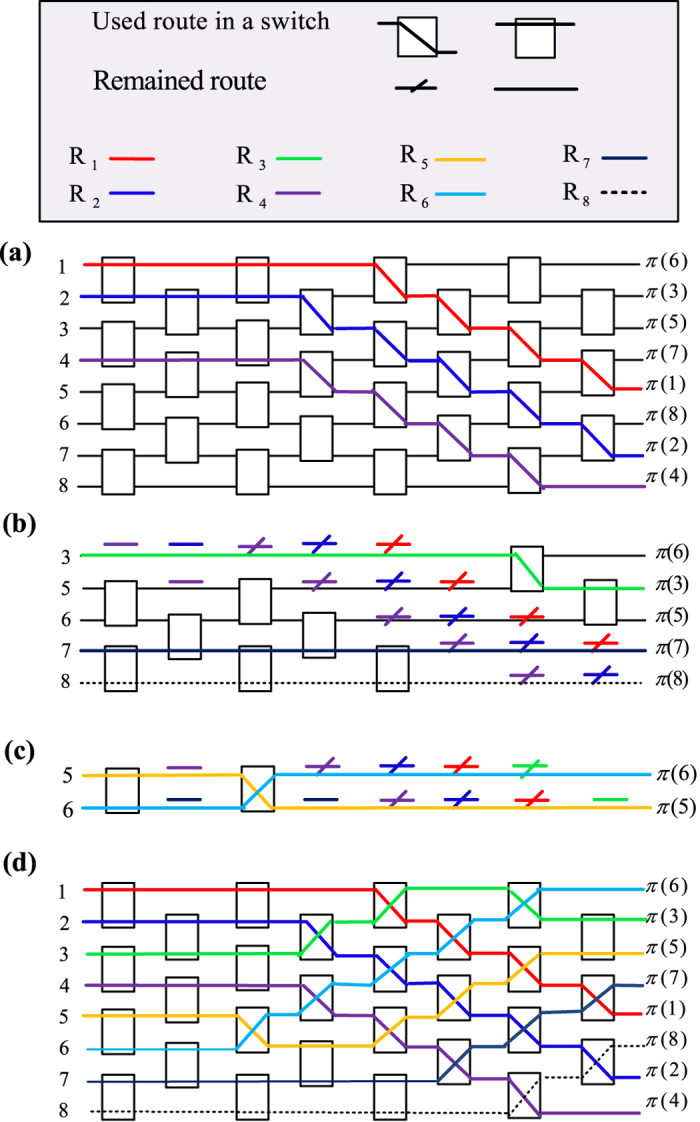
The parallel route-finding algorithm with eight inputs. (**a**) The first-round route with three colors for the inputs 1, 2 and 4 respectively. (**b**) The second-round route with three colors for the inputs 3, 7 and 8 respectively. (**c**) The third-round route with two colors for the inputs 5 and 6 respectively. (**d**) All the routes reconstructed in one network. *R*_*i*_ denotes the *i*-th routing path, *i* = 1, 2, · · · , 8.

In order to realize general quantum network transmission, the primitive 2 × 2 quantum switch may be extended to multiple inputs. Quantum switching networks are analogs of classical switching networks in which classical switches are replaced by quantum switches. These networks are used to switch quantum data among a set of quantum sources and receivers. Similar to classical switch networks^6^,^7^, it easily defines the quantum Benes network^63^. A *N* × *N* quantum Benes network is defined recursively shown in Fig. 6(a). It consists of 2log*N*−1 stages of 2 × 2 quantum switches, with each stage having no more than *N*/2 2 × 2 switches. Similar to the classical Benes network, the quantum Benes network is rearrangeable non-blocking, i.e., for any permutation *π*∈ SN, there exists a setting of the 2 × 2 switches such that *π* can be realized by the network. The simplest classical routing algorithm is the looping algorithm^6^,^7^. The complexity of this algorithm is *O*(*N* log *N*). However, previous route-finding algorithm has not reduced the complexity of distinct pathes. With some pre-coding, a modified algorithm is presented. For *N* input photons 1,2, · · ·, *N*, there are *N* possible outputs. The outputs may be described as *π*(1, 2, · · ·, *N*). Label all lines using *l*_*i*,*j*_ under the matrix order. *i* denotes the input number while *j* denotes the number of links from left to right. For each switch, there are four possible lines. Two lines *l*_*i*,*j*_ and *l*_*j*,*j*_ for each pair (*i*, *j*) denote links for unchanged transmissions. Lines *l*_*i*,*j*_ for each pair (*i*, *j*) denote links for crossly transmissions.

**Algorithm**

(1) The permutation map is given by *π*, where each input *i* is mapped to output *π*(*i*), *i* = 1, · · ·, *N*. Let *S*_1_={*π*(*i*)|*π*(*i*_1_) < *π*(*i*_2_) if *i*_1_ < *i*_2_} and 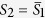 (the complementary set).

(2) Route all the inputs in *S*_1_ in parallel (upward if *π*(*i*) > *i* or downward if *π*(*i*) < *i*, signal by continuing straight across the planar network using *π*(1)−1 stitches, and then forward to last stages. Record the routes by their lines.

(3) Delete the completed paths and associated switches (labeling the remained route in all used switches) from the network [see Fig. 6(b)].

(4) Move the separated upper right corner triangle down and left to reconstruct the planar topology [see Fig. 6(b)].

(5) The remained 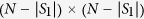 planar network can be routed by recursively applying steps 2–4 [see Fig. 6(c)].

(6) Finally, reconstruct all routes with original links, sequentially. For the *i*-th route, if there are *n*_*i*_ associated switches used by previous *i*-1 routes, it should be connected in order with previous *i*-1 routes [see Fig. 6(d)].

This route-finding algorithm takes only three times in comparison to the seven times using the looping algorithm^6^,^7^. In the first time, 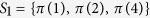, three route pathes may be found in parallel. From the definition of *S*_1_, all these routes have no common links. In the second time, 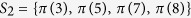. Only three route pathes may be found in parallel because the routes for the outputs *π*(3) and *π*(5) have common links (*π*(3) > *π*(5) and 3 < 5). In the third time, 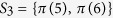. Two route pathes may be found in parallel. The reconstruction algorithm is same to that the looping algorithm^6^,^7^. Generally, this algorithm can reduce at least half time of the looping algorithm^6^,^7^, see SI.

### Single-sided QD system in quantum communication based on two DOFs

Previous results are mainly depended on one DOF, such as the polarization logic gates using the spatial-mode DOF as the assistant^21^,^64^,^65^. With the help of the single-sided QD system, these switches have realized all possible switches of photons with two DOFs. Thus photonic switches show that the independence of the polarization and the spatial mode DOF of photon system. Even if two DOFs may be convert into each other in applications, their conversions may results in failure when they are applied simultaneously. One typical example is derived from the encoding qubit and the error-correction qubit of various algorithms. Moreover, when the different DOFs of different photons are used to encode the same type of information, one should pay attention to their different circuits.

## Additional Information

**How to cite this article**: Luo, M.-X. *et al.* Controlled Photon Switch Assisted by Coupled Quantum Dots. *Sci. Rep.* **5**, 11169; doi: 10.1038/srep11169 (2015).

## Supplementary Material

Supplementary Information
