# Supplementary material for: Controlled Photon Switch Assisted by Coupled Quantum Dots
Source: Sci Rep. 2015 Jun 22;5:11169. doi: 10.1038/srep11169 (PMC4476127; doi:10.1038/srep11169)
Supplement: Supplementary Information [file srep11169-s1.pdf]

Supplemental materials

Controlled Photon Switch Assisted by Coupled Quantum Dots

Ming-Xing Luo, Song-Ya Ma, Xiu-Bo Chen, & Xiaojun Wang

Quantum switch of photons under the electron spin controlling. If the electron spin is used to control the quantum switch, similar circuit may be easily followed. Before the realization of the Toffoli gate on the electron spin and two photons, one hybrid CNOT gate

$$|\uparrow\rangle\langle\uparrow| \otimes (|Rc_1\rangle\langle Rc_1| + |Lc_1\rangle\langle Lc_1|) + |\uparrow\rangle\langle\uparrow| \otimes (|Lc_1\rangle\langle Rc_1| + |Rc_1\rangle\langle Lc_1|) \qquad (1)$$

is used to entangle the general controlling electron spin $\eta_1|\uparrow\rangle+\eta_2|\downarrow\rangle$ and the photon C in the state $|Rc_1\rangle$. Thus the followed circuit is same to that shown in Figures 3 and 4.

Parallel route finding reduction. Consider the order set $\mathcal{S} = (1, 2, 3, \cdots, N)$ and $\overline{\mathcal{S}} = (\pi(i_1), \pi(i_2), \pi(i_3), \cdots, \pi(i_N))$. Here, $\{i_1, i_2, i_3, \cdots, i_N\} = \{1, 2, 3, \cdots, N\}$. If $\pi(1)$ is not the largest number in $\overline{\mathcal{S}}$, there exist another $\pi(i) > \pi(1)$. So, $\{\pi(1), \pi(i)\} \subset \mathcal{S}_1$. Otherwise, i.e, $\pi(1)$ is the largest number in $\overline{\mathcal{S}}$. Now, consider $\pi(2)$ for $\overline{\mathcal{S}} - \{\pi(1)\}$. If all these cases are not satisfied, i.e., $\overline{\mathcal{S}} = (\pi(N), \pi(N-1), \pi(N-2), \cdots, \pi(1))$. Consider $\pi(\lceil N/2\rceil)$ and $\pi(\lceil N/2\rceil + 1)$. Since $\lceil N/2\rceil$ and $\lceil N/2\rceil + 1$ require only one switch, they can be implemented in parallel. So, it has theoretically proved that for any permutation in the symmetric group $\mathcal{S}_N$, $\mathcal{S}_1$ may contain at least two numbers.
